# Human-Computer Interaction and Artificial Intelligence: Advancing Care Through Extended Mind Theory

**DOI:** 10.7759/cureus.74968

**Published:** 2024-12-02

**Authors:** Chris Jacobs, Hans Johnson, Tim Rennie, Jeffrey Lambert, Richard Joiner

**Affiliations:** 1 Psychology, University of Bath, Bath, GBR; 2 Postgraduate Medical Education, Great Western Hospitals NHS Foundation Trust, Swindon, GBR; 3 Digital Health and Care, School of Engineering, Mathematics, and Technology, University of Bristol, Bristol, GBR; 4 Life Sciences, University of Bath, Bath, GBR; 5 Health, University of Bath, Bath, GBR

**Keywords:** artificial intelligence and education, artificial intelligence in medicine, mixed reality (mr), psychiatry and psychology, psychological theory

## Abstract

Artificial intelligence (AI) and mixed reality (MR), within human-computer interaction (HCI), are rapidly redefining areas of healthcare by introducing new approaches to patient care and clinical education. This editorial explores how these technologies, through Extended Mind Theory, enhance mental health treatment and medical training. AI-powered virtual therapists, using natural language processing and predictive analytics, provide accessible, personalized mental health support, allowing for remote and immersive therapy. In MR environments, patients with anxiety, post-traumatic stress disorder (PTSD), or phobias can safely engage in therapeutic exercises, confronting fears in controlled, virtual settings.

In clinical education, AI and MR deliver adaptive, immersive training tools that respond to individual needs, enabling repeated practice in a risk-free environment. These tools improve skills and build confidence by simulating high-stakes scenarios like emergency response, with HCI principles ensuring user-friendly and experiential learning. Ethical considerations, including data security and transparency, are essential as these tools integrate into healthcare. This blend of AI, MR, and HCI redefines healthcare boundaries, extending cognitive and emotional support into virtual spaces, enhancing both patient care and clinical training.

## Editorial

The convergence of artificial intelligence (AI), mixed reality (MR), and human-computer interaction (HCI) is transforming healthcare by creating new avenues for patient care and clinical education. In short, AI enables machines to perform tasks normally requiring human intelligence, MR technology merges physical and digital elements for immersive interaction, and HCI focuses on optimization of user experience in human-technology interfaces. By incorporating Extended Mind Theory, which suggests that cognitive processes can extend beyond the brain to tools and environments, AI and MR applications have the potential to become integral parts of both therapeutic interventions and training tools for healthcare professionals. This editorial explores how these technologies reshape the mental health landscape by enabling virtual therapists and immersive therapeutic environments while also providing powerful educational tools to train clinicians with high fidelity and interactivity.

Extended Mind Theory, introduced by philosophers Andy Clark and David Chalmers, posits that the mind extends into the environment through external tools and devices that support cognitive functions [1]. We have used smartphones in many ways that supplement our brains in computation, handling complex calculations, data storage, and retrieval. In healthcare, this concept can be applied to MR environments powered by AI that can act as cognitive extensions for both patients and clinicians, creating interactive therapeutic and educational experiences. Thus, we can consider the technological interaction as an augmented experience or augmented intelligence. Through HCI design, these systems are optimized for ease of use and adaptability, facilitating real-time adjustments to cater to individual needs and contexts.

Advancing mental health treatment through artificial intelligence

AI's potential to simulate therapy is already evident in mental health, with systematic reviews suggesting it may be effective in reducing symptoms of depression and distress [2]. With the integration of natural language processing (NLP), machine learning, and predictive analytics, AI systems can simulate therapeutic interactions, delivering guided, responsive care. These "virtual therapists" provide personalized support, adapting in real-time based on patient emotional cues and responses [3]. Such systems make mental health therapy accessible to individuals in remote locations, or if they are unable to access mental health therapy, allow patients to engage in exercises like cognitive behavioral therapy (CBT) or exposure therapy within a controlled MR environment. This flexibility not only supports accessibility but also enables a safe space for patients to address complex emotions under structured guidance. These applications extend the therapeutic process, allowing patients to experience the effects of therapy in real-time, reducing the need for frequent in-person visits, and promoting long-term resilience. Furthermore, predictive analytics can use machine learning algorithms to forecast patient trends by triangulating sources of data. This could be an analysis of the patient's clinical record, real-time mood monitoring, and patient feedback through conversation. 

Virtual therapists in MR environments could further be useful for treating anxiety, post-traumatic stress disorder (PTSD), and phobias by allowing patients to confront fears or rehearse coping techniques in a simulated space. For example, a patient with a social phobia might practice interactions with AI-simulated individuals, gradually building confidence through repeated, guided exercises. Recent systematic reviews indicate that virtual reality exposure treatments show positive outcomes for most phobias; however, more rigorous studies are still needed to confirm these findings [4].

**Figure 1:**
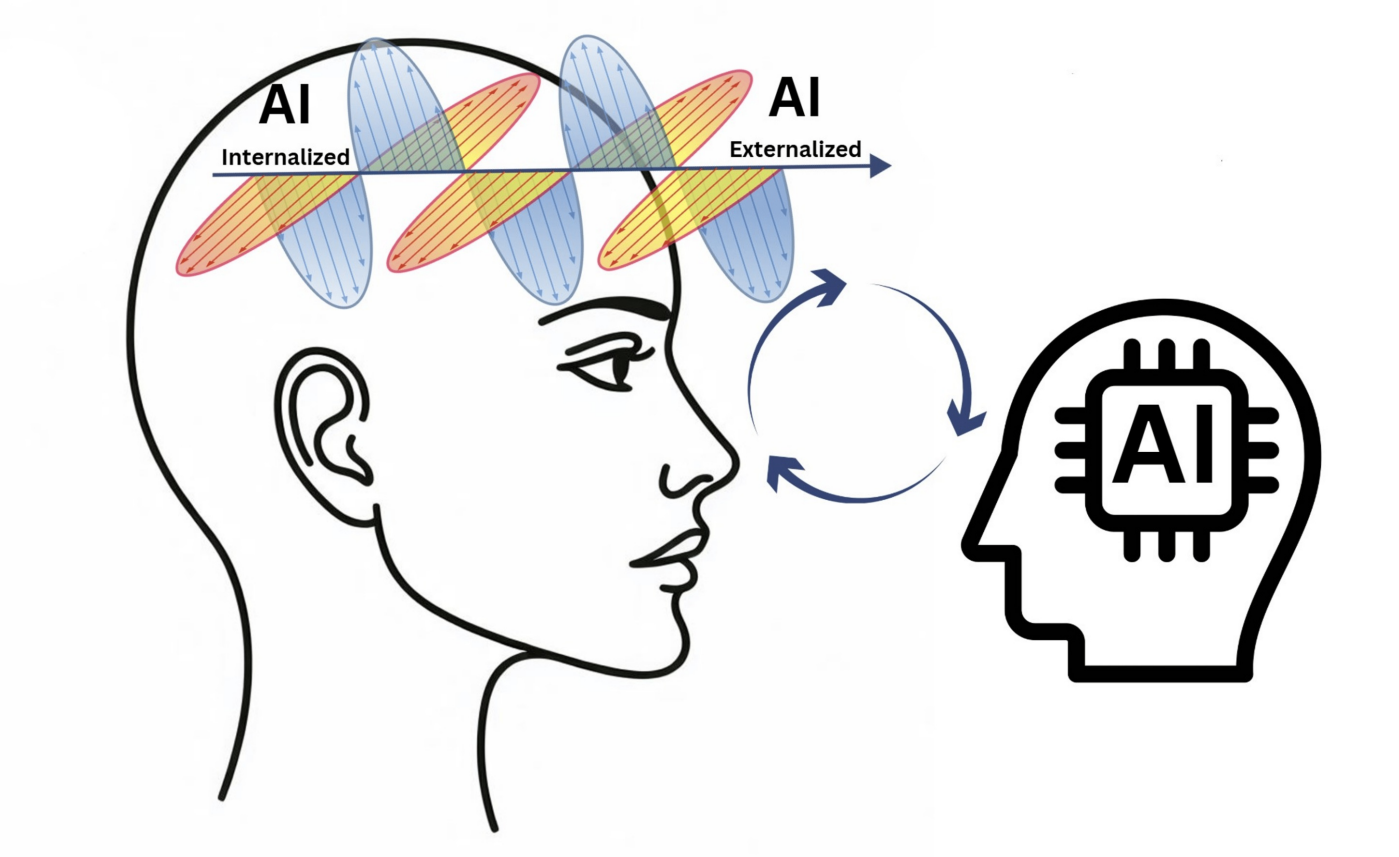
Extended mind theory spectrum of AI embodiment and agency AI: artificial intelligence Image credit: Chris Jacobs

The therapist avatar is simultaneously creating a simulated experience while building on the patient's responses to create a narrative that promotes new thinking. The Extended Mind Theory is a spectrum of active conscious and subconscious, so how much agency we place on ourselves or the AI avatar will inevitably differ depending on the context and conditions of the interaction (Figure 1). On one end of the spectrum we will experience the HCI as a pure thought extension of our mind, and on the other end it will be experienced as if a completely separate person is there. Immersion, presence, and cognitive processes all play a part in how we will experience HCI.

Expanding clinical education with artificial intelligence and mixed reality

Beyond mental health, AI and MR have great potential for clinical education. Medical training, which traditionally relies on simulated patient interactions and cadaveric dissections, can be enhanced by AI MR environments. MR allows medical students to practice complex procedures within immersive, risk-free simulations [5]. AI can be prompted to provide real-time feedback, adjusting difficulty levels based on learner progress, and offers guidance that would otherwise require the constant presence of an instructor. This approach builds a more adaptable and efficient learning environment, tailored to individual learning curves. The mastery of practice is being supplemented by technology that facilitates progress by monitoring a trainee's performance on AI-computed feedback. This can be experienced as sources of internal and external feedback as the AI is purposively directed towards the user; however, the user does not experience the feedback as dissociated from themselves. Hence, Extended Mind Theory proposes that the technology augments the cerebral capacity. 

Virtual reality (VR) simulations in surgical training have been shown to significantly improve procedural skills and reduce anxiety by providing students with repeated practice opportunities [6]. AI further enhances these experiences by personalizing feedback and highlighting potential areas for improvement. In scenarios such as emergency response training, MR environments enable students to practice decision-making under pressure. This realistic simulation fosters confidence and skill retention, essential for handling high-stakes situations. The training impact of MR systems has been observed in areas like resuscitation, intubation, and pediatric care, where complex, high-risk situations can be practiced repeatedly in VR environments.

Furthermore, AI can transcribe a clinical encounter and review the content in relation to inputted metrics. This has the potential to be applied to assessments of clinicians, for example, Observed Structured Clinical Examinations (OSCE). This will aid impartiality in examination and provide a real-time assessment and account, which would directly support any examiner and their findings.

Educators should also understand that increasing numbers of clinicians are integrating AI applications into their work streams. Curriculum design should at best mirror the requirements of the future profession, and incorporating them into medical training could improve proficiency and literacy in AI technologies.

Creating adaptable environments

HCI principles ensure that these AI-driven MR systems are user-centered and responsive to the needs of patients and trainees. Iterative design methods, user experience assessments, and usability testing refine these systems, creating intuitive interfaces that are easy to navigate and reducing cognitive load [7]. For patients in therapeutic settings, this means they can focus on the treatment itself without being overwhelmed by technical complexities. In clinical education, it ensures that students and practitioners can engage fully with the simulation, maximizing learning outcomes.

HCI also enhances adaptability, allowing MR environments to respond to user cues in real time. For instance, a virtual therapist could adjust its approach based on the patient’s emotional state, as detected through NLP and sensor data. Similarly, in a clinical training scenario, AI can adjust task difficulty or offer just-in-time prompts when a trainee struggles. This responsiveness fosters a supportive, dynamic learning experience aligned with Extended Mind Theory by extending cognitive and emotional processes into the MR space.

Ethical and practical considerations for artificial intelligence and mixed reality in healthcare

While AI-driven MR systems offer numerous benefits, they also raise ethical and logistical concerns. For mental health applications, protecting patients and their data is paramount. AI and MR tools handle sensitive personal data, so rigorous protocols around data security and informed consent are essential to maintain trust. Furthermore, robust testing with clinician supervision to ensure safety is necessary. In clinical education, concerns around data privacy and psychological safety exist, particularly as trainees interact with increasingly lifelike simulations or feedback becomes increasingly personalized. HCI should be designed with principles that emphasize transparency, ensuring users understand when they are interacting with AI-driven avatars or simulated environments, which promotes psychological safety. Moreover, if we are to integrate for education, then a quality check on the avatar responses is important if we are rehearsing clinical encounters with the AI being the patient. Fidelity, or realism, of an experience is important for the experiential learning to be congruent and authentic.

In addition, these technologies must account for patient autonomy and ethical engagement. For mental health patients with cognitive impairments, carefully designed MR experiences are required to avoid overwhelming or distressing reactions. Clear safety protocols, customization options, and clinician oversight are critical for minimizing risks while maximizing therapeutic or educational benefits.

Further research evaluating the multiple facets of these technologies is crucial if we are to deploy and embed them into healthcare settings for training or patient care. In particular, safety and efficacy will hopefully be areas of focus for research departments. There also needs to be transparency on system limitations touched on in this review. AI and MR are potential scalable solutions to healthcare needs; however, there is a significant cost to new technology, and integration challenges exist within healthcare structures. Adoption of these technologies requires a change of mindset for a significant number of staff and trainees. Thus, responsible parties whom this challenge falls on should have evidence on the pros and cons of integration in the current form.

Conclusion

The blending of HCI, AI, and MR in healthcare creates a powerful scope for "revisiting reality," supporting both patient care and clinical training through the lens of Extended Mind Theory. In mental health treatment, AI-driven MR environments allow patients to engage with virtual therapists and immersive therapeutic exercises, extending the therapeutic process into their lived environments. In clinical education, these technologies enhance traditional training, creating adaptable, interactive learning spaces that foster skill development and preparedness for high-stakes clinical settings. Revisiting psychological theories helps us to understand the way this new technology is cognitively received and acted upon.

As healthcare systems integrate these tools, the potential for patient-centered, adaptive care grows. With the guidance of HCI principles, these technologies can be designed to be accessible and user-friendly, minimizing cognitive load and creating intuitive, engaging environments.
